# Household Structure and Contraceptive Use in Nigeria

**DOI:** 10.3389/fgwh.2022.821178

**Published:** 2022-05-10

**Authors:** Opeyemi Fadeyibi, Mayowa Alade, Samuel Adebayo, Temitope Erinfolami, Fatimah Mustapha, Saudatu Yaradua

**Affiliations:** ^1^Health, Nutrition, and Population, World Bank, Abuja, Nigeria; ^2^Department of Global Health School of Public Health Boston University, Boston, MA, United States; ^3^Center for Research, Evaluation Resources and Development, Abuja, Nigeria

**Keywords:** contraceptives, family, household composition, polygamy, decision making

## Abstract

**Background:**

Contraceptive use in Nigeria has been consistently low despite its many benefits and several efforts by government and development partners to increase its uptake. According to the Nigeria Demographic and Health Survey, the use of any modern method staggeringly increased from 4 to 12% over 28 years (1990–2018). Studies have identified factors at the individual, household, and societal levels that affect contraceptive use. While studies have also shown that decisions such as contraceptive behavior and acquisition of family skills may vary according to the individual or societal factors, there is a dearth of knowledge on how household structure and composition influence contraceptive use in Nigeria. This article seeks to contribute to the body of knowledge by exploring contraceptive use within the household context.

**Method:**

We used data from the 2018 Nigeria Demographic and Health Survey to examine the relationship between household structure and contraceptive use. We excluded pregnant and non-married women at the time of the survey from the sample and used multinomial regression analysis to examine the likelihood of using traditional or modern methods of contraception.

**Results:**

Results show that having a large household size, and the presence of multiple wives in the household significantly reduces the likelihood of using any method of contraceptive. The result further shows a significant association between household wealth index and contraceptive use as the use of any method increases with household wealth index, with those from richest households being twice as likely as their counterparts in the poorest households to use traditional methods (OR:2.02, *p* < 0.05). Also, women living in households headed by older men (25 and above), households with under 5 children, and those living in rural areas have significantly reduced likelihood of using any method.

**Conclusion:**

This study highlights the dynamics of contraceptive use among married women considering household composition. While our study serves as a primer to understanding contraceptive use in households where a woman and her spouse are usual household members, improved family planning interventions to increase uptake through demand creation will require deeper and more comprehensive work to understand the dynamics among women in more complex household settings.

## Introduction

Family planning is a cost-effective means of reducing maternal morbidity and mortality and achieving sustainable development goals (SDGs) (1–3). However, its use, especially in low and middle-income countries (LMICs), is limited. In the last decade, there has been a rise in modern contraceptives, but still, the number of unmet modern contraceptive needs among women is high (1). In 2019, about 218 million women (about 28%) in developing countries who wanted to prevent pregnancy were not using modern contraception (4, 5). For example, in Nigeria, the unmet need for contraception among currently married women was 19% and 48% among sexually active unmarried women (6). Over the last decade, increased investments in family planning programs increased the rate of contraceptive use from 19 to 62% in developing countries and contributed to an estimated 75% decline in fertility rate (7). However, despite investments and evidence supporting contraceptives, gross inequalities in contraceptive use between and within countries still exist, with rates lowest in LMICs.

Contraceptive use, especially of modern methods is a proven means to reducing maternal and child deaths. It is estimated that about 30% of maternal deaths and 90% of induced abortion-related maternal deaths could be prevented if all women who desired to use contraceptives had access to them (6–10). Additionally, contraception makes significant contributions to reducing infant, neonatal and under-five mortality (7, 10). It is estimated that about 1.8 million child deaths could be averted in developing countries if there is a three-year interval between all pregnancies (11). If children were spaced by a gap of at least 2 years, the infant mortality rates would decrease by about 10% and child mortality by 21% (12). Thus, improving the uptake of contraceptives among women, especially in countries like Nigeria with high maternal and childhood deaths, could significantly improve maternal survival.

Nigeria is the seventh most populous country globally, with an estimated population of about 206 million and an annual population growth of about 2.5% (13). The country's rapid population growth is attributed to a high total fertility rate (TFR) of 5.3 (compared to 2.3 globally) children per woman (13, 14). The high total fertility rate in Nigeria has negative consequences for families and the country. Studies show that by reducing fertility rates, countries reap demographic dividends with the consequent potential of reducing poverty and boosting families' and societies' economic growth and well-being (15). For instance, it is estimated that a reduction in fertility by one child per woman in Nigeria would lead to a 13% increase in GDP per capita within 20 years (16). To achieve demographic dividends and health benefits of contraceptive use, countries must increase their contraceptive prevalence rates. However, the low contraceptive prevalence rate (CPR) in Nigeria persists, stagnating at 17% among currently married women, a rate much lower than the average CPR (36%) in Africa (17). In Nigeria, variation in CPR exists across the country's regions and by sociodemographic characteristics like education, income quintiles, religion, and cultures (17).

Several factors affect contraceptive uptake among women. These include individual-level factors, such as fertility preferences, male child preference, educational level, and exposure to media (6, 18). Also, household or family-level factors such as spousal communication on family planning and autonomy (6, 18) have been associated with contraceptive uptake. Religion and cultural norms at the community level have also been known to influence family planning (6, 18). At the system level too, factors like access to the health facility, availability of methods, and cost of contraceptives influence uptake of family planning methods (6, 18).

In developing countries, the household structure is influenced by religious, cultural, and societal norms (4, 19). These norms vary widely within and between societies due to widespread ethnic diversity, strongly influencing individual choices and behavior. In varying degrees, polygynous unions seem to be the norm in most African societies with about 45% to 55% of women live in polygynous family settings (20, 21). Studies on the effect of family structure on reproductive health and family outcomes show that type of marriage has varied effects on reproductive health. Being in a polygynous union was found to reduce contraceptive use among women in Nigeria (22, 23). Additionally, studies show that the higher the number of living children in a family, the less likelihood of contraceptive use (22, 23). In other settings intrafamilial power dynamics were also found to play a major role in determining the likelihood of contraceptive use. The presence of mothers-in-law in the household for instance was found to influence contraceptive use and other maternal health decisions in places like India, Bangladesh and Mali (24–26).

While studies have shown that decisions such as contraceptive behavior and acquisition of family life skills may vary according to the individual or societal factors, there is a shortage of knowledge on how household structure and composition influence behavior such as contraceptive use in Nigeria. This article seeks to contribute to the body of knowledge on contraceptive use by exploring contraceptive use within the household context. This knowledge will help program implementers to consider contextual household-level factors during the design and development of family planning programs and Social and Behavioral Change Communication interventions.

## Methods

We used data from the 2018 Nigeria Demographic and Health Survey (NDHS) to examine the relationship between household structure and contraceptive use. The study utilized data extracted from the persons' recode (household data) and the individual recode (Women data) files of the 2018 Nigeria Demographic and Health Survey (NDHS). The sampling frame for the survey was the list of enumeration areas (EAs) prepared for the 2006 Population and Housing Census of Nigeria, provided by the National Population Commission. The sample was selected using a stratified two-stage cluster design consisting of 1400 clusters with enumeration areas (EAs) as the primary sampling units for the first stage. A sample of 42,000 households was selected for the survey, with a 99.3 and 99.5% response rate in urban and rural areas, respectively.

For this study, we only included women whose husbands were reported to be the household heads or who were household heads themselves. Also, we excluded unmarried and currently pregnant women from the study sample. Unmarried women were excluded because the types of household composition that are important to the study, most especially those defining a woman's relationship with her partner's family members, could not be established with the available data. In the same vein, currently, pregnant women were excluded because they are not expected to be using contraception at the time of the survey. Eligible women were thereafter linked across the household file, thus creating a new dataset for the study, with a total of 21,467 married women who were either household heads or spouse to the head of the household, to be able to measure how the presence of in-laws and other relatives in the household affect the use of contraceptives. We examined the relationship between the current use of any method of contraception and household variables such as household structure and composition, physical facilities, characteristics of the head of household and adjusted for the individual woman characteristics. We modeled the outcome using multinomial logistic regression.

### Variable Measurements

**Outcome variable:** The outcome variable for the study was current contraceptive use. This was defined as the use of any form of contraception at the time of the survey by married women 15–49 who are not currently pregnant and had at least a child. The variable was categorized into three; not using any method categorized “0,” using any traditional method “1,” and using any modern method “2.” We measured the outcome variable as a nominal variable, thus assuming that no category is greater in importance than the other. We then used multinomial logistic regression to predict the probabilities of the different possible outcomes of using a traditional method or a modern method. The use of any modern methods was defined as the use of one of the following methods: female sterilization, male sterilization, pill, intrauterine contraceptive device (IUD), injectables (Depo-Provera), implants (Norplant), female condom, male condom, diaphragm, contraceptive foam and contraceptive jelly, lactational amenorrhea method (LAM), standard days method (SDM). The use of any traditional method, however, implies the current use of one of the following methods: periodic abstinence (rhythm, calendar method) and withdrawal (coitus interruptus).

**Explanatory variables** include household composition, physical facilities, and women's individual characteristics. Household composition measures household demographics such as the: i) presence of children below age 5 (1 if yes and 0 otherwise); ii) number of male children; iii) co-residence with in-laws (brothers, sisters, and/or parents of the partner): iv) older household heads than respondents (1 if yes, 0 otherwise); v) presence of co-wives and vi) household wealth index (quintiles). Women's individual-level factors were used as the intervening variable and include age, education, number of children, decision-making power, number of male children, presence of co-wives, and employment status.

### Statistical Analysis

Univariate analysis involving frequency and percentage distributions was used to report the prevalence of contraceptive use and household characteristics of respondents. Chi-square test of association and multinomial logistic regression was utilized to examine the statistical associations between the outcome and the explanatory variables at 95% confidence intervals. Multicollinearity was evaluated with simple correlations among the independent variables. The multivariate model examined the relationship between selected household variables, woman individual-level variables, and the current use of any method. All analyses were done using STATA software version 16.1.

## Results

### Description of Respondents' Household and Background Characteristics

Table 1 presents the description of the respondent's household characteristics. The results show that two-thirds of the households were headed by men 25–49 years (67.2%), while 32.7% of household heads did not have any education. About 8 in 10 households had at least one under-5 child. Almost half of the households had a minimum of 5 members with slightly above one-quarter (26.6%) of household heads having multiple wives. The extended family living arrangement in which an in-law lives with the family was not widespread as only 5.3% reported living in households with such living arrangements. There were more women in rural households compared to those in urban (60 vs. 40% respectively).

**Table 1:** Description of the household characteristics of the study sample.

	**Weighted N (21,886)**	**%**
**Age of household head**		
Below 25 years	915	4.2%
25–49 years	14,715	67.2%
50 and above	6,256	28.6%
**Highest educational level attained (HH head)**		
No education	7,154	32.7%
Primary	4,203	19.2%
Secondary	6,864	31.4%
Higher	3,642	16.6%
**Household Size**		
5 and below	10,169	46.5%
Above 5	11,717	53.5%
**Presence of children under 5 in household**		
No	4,661	21.3%
Yes	17,225	78.7%
**Multiple wives present in household**		
No	16,055	73.4%
Yes	5,830	26.6%
**Presence of in-laws in household**		
No	20,720	94.7%
Yes	1,166	5.3%
**Wealth index**		
Poorest	4,645	21.2%
Poorer	4,730	21.6%
Middle	4,068	18.6%
Richer	4,085	18.7%
Richest	4,358	19.9%
**Type of place of residence**		
Urban	8,771	40.1%
Rural	13,115	59.9%

The results in Table 2 further show that almost half of the women had no education (46.9%). While only 5.2% had never given birth, 42% of the women had 4 or more children. About 72% of women in the sample reported that they made decisions alone regarding their earnings and 19.9% made this decision jointly with their spouse. Involvement in domestic decision-making was low as 37.3 and 16.5% of women were not involved in decision-making at all or had low decision-making power respectively. Seven in ten women were working while 32.1% had their co-wives present in their household. Only 14% of women in the sample used a modern method of contraceptive.

**Table 2:** Percentage distribution of women by background characteristics.

	**Weighted N (21,886)**	**%**
**Current Age**		
Below 25 years	4,194	19.2%
25–34 years	8,359	38.2%
35–49 years	9,332	42.6%
**Highest educational level**		
No education	10,256	46.9%
Primary	3,523	16.1%
Secondary	6,085	27.8%
Higher	2,022	9.2%
**Number of Children**		
Never given birth	1,140	5.2%
1–4 children	11,555	52.8%
4 or more children	9,190	42.0%
**Person who usually decides how to spend respondent's earnings**		
Respondent alone	9,869	71.8%
Respondent and husband/partner	2,733	19.9%
Husband/partner alone	1,142	8.3%
Someone else	7	0.1%
**Domestic decision making**		
No decision	8,157	37.3%
Low	3,609	16.5%
Middle	3,013	13.8%
High	7,106	32.5%
**Respondent currently working**		
No	6,326	28.9%
Yes	15,560	71.1%
**Husband has other wives**		
No other wives	14,856	67.9%
Other wives present	7,030	32.1%
**Current use of FP by method type**		
No method	17,630	80.6%
Traditional method	1,160	5.3%
Modern method	3,096	14.1%

### Respondents' Contraceptive Behavior by Household and Individual Characteristics

The results in Tables 3, 4 show the distribution of women's contraceptive use by household and individual characteristics, respectively. Although contraceptive use was generally low among the study sample, the household variables considered in this study significantly affect both use and type of method. As shown in Table 3, contraceptive use was significantly associated with selected household characteristics (*p* < 0.001). The proportion of women in large households (5 or more) using modern contraceptives was slightly higher than those who used traditional methods, while those in polygamous households using modern method is almost twice those using traditional method (12.3 vs. 6.9%). Use of any method increased significantly with wealth quintile, age, and level of education of household heads (up to secondary school). Place of residence and presence of under-5 children were also significantly associated with contraceptive use.

**Table 3:** Bivariate analysis of women's household characteristics and contraceptive use.

	**No method**	**Traditional method**	**Modern method**	**Chi-square**
**Household size**				
5 or less	7,945 (81%)	478 (49.9%)	1,383 (46.8%)	10.2*p* = 0.006
Above 5	9,641 (82.5%)	480 (50.1%)	1,570 (53.2%)	
**Multiple wives in household**	12,330 (78%)	66 (6.9%)	363 (12.3%)	599.5, *p* = 0.000
**Presence of In-law**	973 (79.5%)	75 (7.8%)	176 (6%)	9.4, *p* = 0.009
**Wealth index combined**				
Poorest	4,670 (93.9%)	46 (4.8%)	259 (8.8%)	1,748.1 *p* = 0.000
Poorer	4,226 (89.6%)	85 (8.9%)	407 (13.8%)	
Middle	3,442 (82.6%)	155 (16.2%)	570 (19.3%)	
Richer	2,915 (73.4%)	267 (27.9%)	788 (26.7%)	
Richest	2,333 (63.6%)	405 (42.3%)	929 (31.5%)	
**Age of HH head**				
Below 25 years	816 (91.2%)	15 (1.6%)	64 (2.2%)	153.5 *p* = 0.000
25–49 years	11,350 (79.6%)	710 (74.1%)	2,194 (74.3%)	
50 and above	5,420 (85.4%)	5,420(30.8%)	233(24.3%)	695 (23.5%)
**Highest educational level HH Head**				
No education	6,666 (93.9%)	78 (8.1%)	352 (11.9%)	1,261.4 *p* = 0.000
Primary	3,490 (82.8%)	194 (20.3%)	531 (18%)	
Secondary	4,922 (73.9%)	438 (45.7%)	1,300 (44%)	
Higher	2,498 (71.1%)	246 (25.7%)	767 (26%)	
**Type of place of residence**				
Urban	5,445 (71.6%)	605 (63.2%)	1,553 (52.6%)	856.0 *p* = 0.000
Rural	12,141 (87.4%)	353 (36.8%)	1,400 (47.4%)	
**Presence of Children Under 5 children in HH**	13,656 (81%)	782 (81.6%)	2,423 (82.1%)	34.9802 *p* = 0.000

**Table 4:** Bivariate analysis of women's individual characteristics and contraceptive use.

	**No method**	**Traditional method**	**Modern method**	**Chi-square**
**Respondent's current age**				
Below 25 years	3,658 (89.9%)	83 (8.7%)	328 (11.1%)	224.7603, *p* = 0.000
25–34 years	6,439 (79.8%)	396 (41.3%)	1,235 (41.8%)	
35–49 years	7,489 (80%)	479 (50%)	1,390 (47.1%)	
**Highest educational level**				
No education	9,482 (93.7%)	102 (10.6%)	538 (18.2%)	2,103.2523, *p* = 0.000
Primary	2,844 (78.6%)	178 (18.6%)	595 (20.1%)	
Secondary	4,137 (69.8%)	472 (49.3%)	1,322 (44.8%)	
Higher	1,123 (61.5%)	206 (21.5%)	498 (16.9%)	
**Number of Children**				
Never given birth	1,086 (98.6%)	2 (0.2%)	13 (0.4%)	252.9655, *p* = 0.000
1–4 children	8,890 (79.8%)	596 (62.2%)	1,660 (56.2%)	
4 or more children	7,610 (82.3%)	360 (37.6%)	1,280 (43.3%)	
**Person who usually decides how to spend respondent's earnings**				
Respondent alone	7,373 (80.8%)	402 (55.8%)	1,346 (65.4%)	237.5302, *p* = 0.000
Respondent and husband/partner	1,933 (69.5%)	271 (37.6%)	576 (28%)	
Husband/partner alone	1,099 (85.9%)	46 (6.4%)	135 (6.6%)	
Someone else	5 (83.3%)	1 (0.1%)	0 (0%)	
**Participation in Decision Making**				
None	7,504 (90.8%)	132 (13.8%)	631 (21.4%)	1,112.8696 *p* = 0.000
Low	2,855 (85.1%)	85 (8.9%)	415 (14.1%)	
Middle	2,200 (78.2%)	129 (13.5%)	484 (16.4%)	
High	5,027 (71.2%)	612 (63.9%)	1,423 (48.2%)	
**Respondent currently working**	12,008 (79%)	802 (83.7%)	2,394 (81.1%)	281.4425 *p* = 0.000
**Partner has other wives**				
No other wife	11,470 (78.3%)	834 (87.1%)	2,339 (79.2%)	393.3206 *p* = 0.000
Other wives present	6,116 (89.2%)	124 (12.9%)	614 (20.8%)	
**Number of Male Children**				
None	3,592 (89.6%)	115 (2.9%)	303 (7.6%)	
One male child	4,709 (81.1%)	290 (5.0%)	807 (13.9%)	213.16 *p* = 0.000
More than one male child	9,285 (79.5%)	553 (4.7%)	1,843 (15.8%)	

Women's individual characteristics were also significantly associated with contraceptive use (Table 4). The percentage of women in older age groups who were using modern (41.8% among 25–34 years and 47.1% among 35–49 years) or traditional methods (41.3% among 25–34 years and 50% among 35–49 years) was higher compared to younger women. Contraceptive use of any type increased with the level of education up to secondary school and is highest among women who had 1–4 children (62.2% for traditional methods, and 56.2% for modern methods). Contraceptive use was significantly higher among women who made decisions alone regarding their earnings (55.8% for traditional method, 65.4% for modern method), and those who jointly decided with their spouse (37.6% for traditional method, 28% for modern method) compared with those who were not involved in deciding how money was spent. Likewise, the percentage of women who used contraception was highest among those whose participation in decision-making was high. Women who are in polygamous unions (other wives present) reported less use of any method, while those with no male child have significantly higher rate of non-use of any method compared with those who have at least 1 male child.

### Results of Multivariate Analysis

Table 5 presents the results of multinomial regression estimating the likelihood of using the traditional (model 1) or modern method (model 2) vs. not using any method in relation to women's household characteristics. The result shows that while having a large household size significantly increased the likelihood of using any method, the presence of multiple wives significantly reduced use of any method of contraception. The result also shows a significant association between household wealth and contraceptive use. Use of any method increased with household wealth, with those from the richest households being thrice as likely to use the modern method (OR:3.15, *p* < 0.05) compared to those from the poorest. Women living in households headed by older men (25 and above), households with under five children have a higher likelihood of using either the modern or traditional method. Those living in rural areas had a significantly reduced likelihood of using any method. As the education of the household head progresses, it increases the likelihood of a woman using a modern method and reduces the likelihood of using a traditional method.

**Table 5:** Multinomial logistics regression reporting adjusted odds ratio for household composition variables and method use.

	**Traditional method (Model 1)**		**Modern method (Model 2)**	
	**OR**	**95% CI**	**OR**	**95% CI**
Household size above 5	1.48^***^	[1.28–1.72]	1.55^***^	[1.42–1.70]
Multiple wives in Household	0.26^***^	[0.20–0.34]	0.42^***^	[0.37–0.47]
Presence of in-law	1.10	[0.85–1.42]	0.81^*^	[0.68–0.97]
**Household wealth index**				
Poorest (Ref)	1			
Poorer	1.59^*^	[1.10–2.30]	1.36^***^	[1.15–1.61]
Middle	2.60^***^	[1.83–3.69]	1.78^***^	[1.51–2.10]
Richer	4.26^***^	[3.00–6.05]	2.37^***^	[2.00–2.82]
Richest	7.52^***^	[5.25–10.79]	3.15^***^	[2.62–3.78]
**Age of HH head**				
15–24 years (Ref)	1			
25–49 years	1.79^*^	[1.05–3.03]	1.53^**^	[1.17–2.00]
50 and above	1.75^*^	[1.01–3.03]	1.33^*^	[1.00–1.76]
**Education of HH head**				
None (Ref)				
Primary	2.46^***^	[1.85–3.25]	2.01^***^	[1.73–2.33]
Secondary	2.40^***^	[1.82–3.16]	2.61^***^	[2.27–3.01]
Higher	1.95^***^	[1.45–2.63]	2.56^***^	[2.18–3.00]
**Residence**				
Urban	1			
Rural	0.66^***^	[0.56–0.77]	0.78^***^	[0.71–0.86]
Presence of under5 child in HH	1.82^***^	[1.51–2.18]	1.59^***^	[1.42–1.78]

*** *p < 0.001*,

** *p < 0.01*,

* *p < 0.05*.

Table 6 shows the results of multinomial logistic regression analysis estimating the effect of individual women's characteristics on contraceptive use. Two models are presented in the table. Model 3 presents the adjusted odds ratio of respondents using a traditional method compared to those not using at all, while model 4 presents the adjusted odds of using a modern method compared with not using any method. The result shows that the odds of using any method is significantly higher at older ages compared to the reference group, and then reduced later with age (OR: 1.58, *p* < 0.001 for 25–34; and OR: 1.45, *p* < 0.05 for 35–49) for traditional methods. The same pattern is observed for modern methods (OR: 1.31, *p* < 0.01, and OR: 1.09, for 25–34 and 35–49 respectively). On the contrary, the odds of using any method increased with levels of education among the women. Although the likelihood of contraceptive use increased with the number of children a woman had, the relationship is not significant among those who used traditional methods. The likelihood of using a traditional method was significantly higher among women who reported that someone else decided on their earnings for them (OR: 14.58, *p* < 0.05) and significantly lower among women using modern methods who reported joint decisions regarding earnings (OR: 0.00, *p* < 0.05). Contraceptive use, especially traditional methods, increased significantly with an increase in woman's participation in household decision-making. Women with low or middle-level participation in the household decision are twice more likely to use any method. Examining the effect of having a male child on the use of contraceptives, women with at least a male child are more likely to use either modern or traditional methods, while the odds increase as the number of male children per woman increases.

**Table 6:** Multinomial logistics regression reporting adjusted odds ratio for women's individual variable and method use.

	**Traditional method (Model 3)**		**Modern method (Model 4)**	
	**OR**	**95% CI**	**OR**	**95% CI**
**Age**				
15–24	1			
25–34	1.58^**^	[1.12–2.22]	1.31^**^	[1.07–1.59]
35–49	1.45^*^	[1.02–2.07]	1.09	[0.89–1.34]
**Level of education**				
No education	1			
Primary	4.06^***^	[2.94–5.60]	3.37^***^	[2.85–3.97]
Secondary	6.65^***^	[4.91–9.01]	5.29^***^	[4.53–6.18]
Higher	9.15^***^	[6.52–12.83]	7.44^***^	[6.16–8.99]
**Number of children**				
Never given birth	1			
less than 4 Children	13,806,064.57	[0.00 -.]	16.61^***^	[6.12–45.09]
4 or more Children	14,098,679.81	[0.00 -.]	18.31^***^	[6.70–50.05]
**Who decides how to spend respondent's earnings**				
Respondent alone	1			
Respondent and husband/partner	1.18	[0.99–1.40]	0.98	[0.86–1.10]
Husband/partner alone	1.05	[0.75–1.45]	0.79^*^	[0.65–0.96]
Someone else	14.58^*^	[1.38–154.41]	0.00	[0.00 -.]
**Household decision Making Participation**				
No decision	1			
Low	1.76^**^	[1.23–2.52]	1.43^***^	[1.20–1.71]
Middle	2.00^***^	[1.42–2.83]	1.68^***^	[1.41–2.00]
High	3.14^***^	[2.36–4.18]	1.59^***^	[1.37–1.85]
**Employment status**				
Not employed	1			
Currently employed	1.12	[0.67–1.89]	0.94	[0.71–1.24]
**Presence of other wives**				
No other wives	1			
Other wives present	0.52^***^	[0.40–0.66]	0.82^**^	[0.72–0.94]
**Have male child**				
No male child				
Only one male child	1.57^**^	[1.19–2.08]	1.68^***^	[1.39–2.02]
More than one male child	1.94^***^	[1.47–2.55]	2.31^***^	[1.92–2.77]

*** *p < 0.001*,

** *p < 0.01*,

* *p < 0.05*.

## Discussion

The government of Nigeria and development partners in the country are making efforts to address supply-side issues related to contraceptive use and ensure that it becomes more widely acceptable both for spacing and limiting the number of children among women of childbearing age. Studies have shown however that demand-side challenges for family planning persist across the country and may be more difficult to tackle (27–32). This article is an attempt to contribute to the body of knowledge in identifying some household characteristics that may contribute to the demand-side challenges for family planning acceptance and use. The study found that the educational level of a household head is significantly associated with contraceptive use. Likewise, a woman's level of education increases the likelihood of using any method of contraception. The likelihood of using a traditional method increases more among women than the use of a modern method. Education and autonomy (measured in terms of a woman's level of participation in household decision-making) were both found to have positive effects on contraception. In this study, the level of participation in decision-making is significantly associated with contraceptive use and women who participate more in decision making are likely to use a traditional method. The study further found that the presence of other wives in the household significantly reduces contraceptive use. It is common practice that in a polygamous household, women compete among other things for their husband's attention. They also compete to have the highest number of children especially male children as this may indicate their right-standing in the husband's family. Hence, women in polygamous households who have not met their fertility goals or desires may not use contraception of any type. Audu et al. found these assertions to be true in their study population where they found that women in polygamous households were significantly less likely to use contraception if they are 35 years or more, have no male child, have 3 or more daughters, or live in rural areas (33). Similarly, Adewole et.al. also identified polygamy as one of the factors influencing fertility and contraceptive use (34).

The presence of in-laws in the study population's households significantly reduces the use of either the traditional or modern contraceptive methods. This finding is an indication that the extended family system which is common in many developing countries including Nigeria may have a strong influence on reproductive health decision-making such as the use of contraception. Mothers and mothers-in-law are strongly believed to influence fertility decisions as their presence may put pressure on their daughters or daughter-in-law in different ways (35–37). This is especially likely when a couple does not have any male children yet. In settings such as India, the presence of mother-in-law has been found to have mixed effects on contraceptive use. For example, Char et al. found that although mothers-in-law have a strong influence on family decisions regarding activities, they do not have a similar influence on the use of family planning among the young couple they studied in a rural Indian community (26). In Nigeria, there is a dearth of knowledge on how the extended family influences the reproductive decisions of married men and women. Family size is known to affect contraceptive use, especially in LMICs. Women who already have their desired number of children, and those who are satisfied with the sex composition of their children are more likely to use contraception (38, 39). In our study, however, we found that living in large households (more than 5 persons) significantly reduces contraceptive use. This finding requires further research. Finally, the study also found that being involved in decision-making about how to spend earnings is significantly associated with contraceptive use.

## Conclusion

This study highlights the dynamics of using traditional and modern contraceptive methods among married women living in households whose compositions are somewhat complex. While our study serves as a primer to understanding contraceptive use in households where a woman and her spouse are usual household members, improved family planning interventions to improve uptake in terms of demand creation will require deeper and more comprehensive work to understand the dynamics among other women in other types of more complex household settings.

### Program/Policy Implication

This knowledge is useful in designing family planning programs and SBCC considering contextual household-level factors.

### Limitations to the Study

The sample was limited to women who are heads of households or married to the household head. Thus, unmarried sexually active women in the sampled households were excluded. Given the contributions of unmarried sexually active women, especially adolescents to the high fertility regime in Nigeria, and the social and cultural norms that discourage contraceptive use by this population subgroup, it will be interesting to understand how household structure affects their contraceptive behavior.

## Data Availability Statement

Publicly available datasets were analyzed in this study. This data can be found here: https://dhsprogram.com/Countries/Country-Main.cfm?ctry_id=30c=NigeriaCountry=Nigeriacn=r=1.

## Author Contributions

OF conceptualized the research idea, developed the research plan, and interpreted the results. MA reviewed the literature and contributed to the discussion. SA and TE conducted all statistical analysis and contributed to writing up the results. FM and SY reviewed the draft manuscript. All authors contributed to the discussion and review of the final manuscript. All authors contributed to the article and approved the submitted version.

## Conflict of Interest

The authors declare that the research was conducted in the absence of any commercial or financial relationships that could be construed as a potential conflict of interest.

## Publisher's Note

All claims expressed in this article are solely those of the authors and do not necessarily represent those of their affiliated organizations, or those of the publisher, the editors and the reviewers. Any product that may be evaluated in this article, or claim that may be made by its manufacturer, is not guaranteed or endorsed by the publisher.
